# Analysis of human metabolism by reducing the complexity of the genome-scale models using redHUMAN

**DOI:** 10.1038/s41467-020-16549-2

**Published:** 2020-06-04

**Authors:** Maria Masid, Meric Ataman, Vassily Hatzimanikatis

**Affiliations:** 1 Laboratory of Computational Systems Biotechnology, École Polytechnique Fédérale de Lausanne (EPFL), Lausanne, Switzerland; 2 Computational and Systems Biology, Biozentrum, University of Basel, Basel, Switzerland

**Keywords:** Metabolic engineering, Cancer metabolism, Metabolic pathways, Computational models, Data integration

## Abstract

Altered metabolism is associated with many human diseases. Human genome-scale metabolic models (GEMs) were reconstructed within systems biology to study the biochemistry occurring in human cells. However, the complexity of these networks hinders a consistent and concise physiological representation. We present here redHUMAN, a workflow for reconstructing reduced models that focus on parts of the metabolism relevant to a specific physiology using the recently established methods redGEM and lumpGEM. The reductions include the thermodynamic properties of compounds and reactions guaranteeing the consistency of predictions with the bioenergetics of the cell. We introduce a method (redGEMX) to incorporate the pathways used by cells to adapt to the medium. We provide the thermodynamic curation of the human GEMs Recon2 and Recon3D and we apply the redHUMAN workflow to derive leukemia-specific reduced models. The reduced models are powerful platforms for studying metabolic differences between phenotypes, such as diseased and healthy cells.

## Abstract (web summary)

The complexity of genome-scale metabolic networks (GEMs) hinders their application in specific physiological contexts. Here, the authors introduce a framework to reduce thermodynamically curated GEMs to the subnetworks of interest and demonstrate its application by deriving leukemia-specific models.

## Introduction

An altered metabolism is a hallmark of several human diseases, such as cancer, diabetes, obesity, Alzheimer’s, and cardiovascular disorders^1,2^. Understanding the metabolic mechanisms that underlie this reprogramming guides the discovery of new drug targets and the design of new therapies. To this effect, tremendous efforts are now being made to use the large amounts of now-available multi-omics experimental data to gain insight into the metabolic alterations occurring in different phenotypes. Unfortunately, current mathematical models can be too complex for this analysis, rendering them too cumbersome to employ for many systems biology studies.

In the field of systems biology, genome-scale metabolic models (GEMs) integrate available omics data with genome sequences to provide an improved mechanistic understanding of the intracellular metabolism of an organism. GEMs have been reconstructed for a large diversity of organisms spanning from bacteria to mammals^3–5^ and are valuable tools for studying metabolism^6,7^. The mathematical representation of GEMs through the stoichiometric matrix^7^ is amenable to methods such as flux balance analysis (FBA)^8^ and thermodynamic-based flux balance analysis (TFA)^9–13^, which ensure that the modeled metabolic reactions retain feasible concentrations and their directionalities obey the rules of thermodynamics, to predict reaction rates and metabolite concentrations when optimizing for a cellular function, such as growth, energy maintenance, or a specific metabolic task. Additionally, GEMs can be used for gene essentiality^14^, drug off-target analysis^15^, metabolic engineering^16–18^, and the derivation of kinetic models^19–22^.

The first human GEM was reconstructed in 2007^23,24^. Since then, the scientific community has been working to develop high-quality human GEMs, including HMR 2.0^25^, Recon 2^26^, Recon 2.2^27^, and Recon 3D^28^. The human GEMs used for the analysis in this article are Recon 2 and Recon 3D. Recon 2 is composed of 7440 reactions with 4821 of them associated to 2140 genes, and 2499 unique metabolites across seven compartments: cytosol, mitochondria, peroxisome, Golgi apparatus, endoplasmic reticulum, nucleus, and lysosome. Recon 3D is the latest consensus human GEM. It is an improved more comprehensive version of the previous GEMs consisting of 10,600 reactions, with 5938 of them associated with 2248 genes, and 2797 unique metabolites compartmentalized as Recon 2 with an additional compartment for the mitochondria intermembrane space.

Human GEMs reconstruct the metabolic reactions occurring in several human cell types. However, a given cell type only leverages a portion of these reactions. This motivates the development of methods to generate context-specific metabolic models that can be used to study the differences in metabolism for different cell types^29^, for healthy and diseased cells^30,31^, and for cells growing under diverse extracellular conditions. Some examples of such methods are (1) GIMME^32^, mCADRE^33^, and tINIT^34^ to reconstruct tissue-specific models based on *omics* data and a set of tasks or a specific objective function; (2) redGEM–lumpGEM^35,36^ to reconstruct models around a specific set of subsystems of interest for the study; and (3) iMM^37,38^ to characterize the extracellular medium and the metabolites that are essential for growth under each condition. Context-specific metabolic models have been extensively used to understand the differences in metabolism between cancer cells and their healthy counterparts^39–45^.

In this article, we present redHUMAN, a workflow to reconstruct thermodynamic-curated reductions of the human GEMs Recon 2 and Recon 3D. We integrate the thermodynamic properties of the metabolites and reactions into the GEMs and use redGEM–lumpGEM to reconstruct reduced models around specific subsystems. Furthermore, we introduce redGEMX, a method to identify the pathways required to connect the extracellular compounds to a core network. redGEMX guarantees that the reduced models have all the feasible pathways that consume and produce the components of the extracellular environment of the cell. Finally, we use metabolic data for leukemia as an example of how to integrate experimental data to derive disease- and tissue-specific metabolic models.

## Results

### Overall workflow

**Fig. 1:**
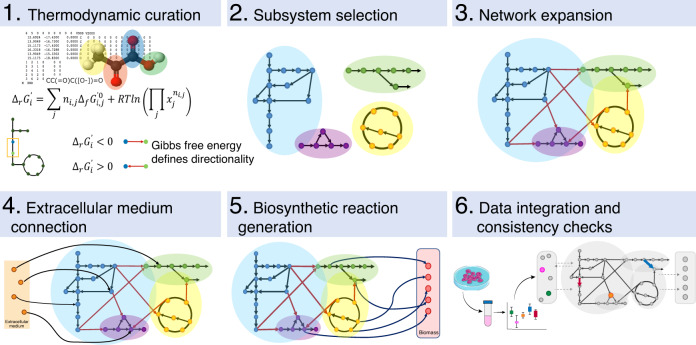
redHUMAN workflow. (1) Thermodynamic curation: the Gibbs free energy of compounds and reactions are estimated and used to define the reaction directionality. (2) Subsystem selection: the subsystems relevant for the study are selected. (3) Network expansion: the initial subsystems are connected using reactions from the GEM to generate a core network. (4) Extracellular medium connection: the pathways that connect the extracellular medium components to the core network are identified. (5) Biosynthetic reaction generation: the pathways required to produce the biomass building blocks are classified. (6) Data integration and consistency checks: experimental values are integrated and the model is verified through consistency checks.

In order to generate reduced models from human GEMs, we developed redHUMAN, a six-step workflow that can be applied to any GEM or desired model system. The overall workflow is briefly described here and shown in Fig. 1, and the details of each step in its application to the human GEMs Recon 2 and Recon 3D to generate thermodynamic-curated reductions are provided in the subsequent sections. For the workflow, the thermodynamic information for compounds and reactions, which is assembled from earlier studies or estimated using established group contribution methods, is first integrated into the GEM. Second, the subsystems, or families of pathways with a specific functional role for a biological process, are selected based on the objectives of the specific study. These pathways are explicitly represented and constitute the core of the reduced model. For example, when studying cancer metabolism, this can include reported subsystems that are deregulated in cancer cells in addition to the standard central carbon pathways. Third, these subsystems are expanded using reacti\ons from the GEM to create a connected core network. In this step, we include every reaction that connects core metabolites and that is not a member of the formal definition of the selected subsystems in the core model. In steps four and five, we include the shortest pathways to connect the extracellular metabolites from the defined medium as well as the shortest pathways to generate the biomass components from the core network. These steps guarantee that the model has all pathways that are essential for survival and growth of the cells based on the availability of nutrients. In the sixth step, experimental data for a specific physiological state is integrated in the model, and the final model is verified through checks that ensure the consistency of the reduced model with the original GEM.

### Thermodynamic curation of the human GEMs (Step 1)

**Fig. 2:**
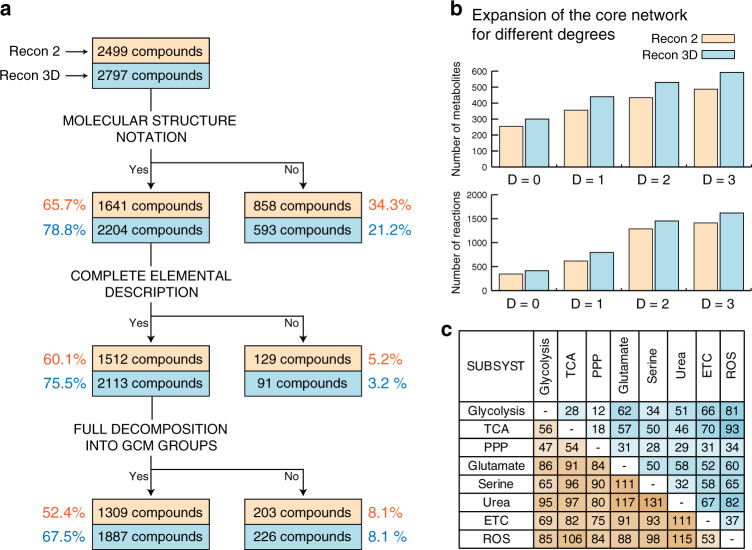
Thermodynamic curation of human GEMs. **a** Thermodynamics for the unique compounds in Recon 2 (orange) and Recon 3D (blue). The percentage is relative to the total number of unique compounds. **b** Size of the core network when the expansion is performed for different degrees. **c** Number of reactions that pairwise connect the subsystems for Recon 2 (values below the diagonal) and Recon 3D (values above the diagonal) for degree *D* = 1.

We first determine the directionality of the chemical reactions of the network, which is directly associated with their corresponding Gibbs free energy. The Gibbs free energy of a reaction can be estimated from the thermodynamic properties of its reactants and products. Therefore, we curated the GEMs Recon 2 and Recon 3D (see “Methods”) and integrated the thermodynamic properties for 52.4% of the 2499 unique metabolites from Recon 2 and 67.5% of the 2797 unique metabolites from Recon 3D (Fig. 2a and Supplementary Data 1). Three main reasons prevented the estimation of the thermodynamic properties of the metabolites: (1) an unknown molecular structure (SMILE), (2) an incomplete elemental description (for example, an R in the structure), and (3) groups in the structure for which an estimated free energy does not exist (for example, >N^−^ group). We observed that as the number of metabolites increases from Recon 2 to Recon 3D, the percentage of thermodynamic coverage increases as well. This is due to the improved annotation of the metabolite structures in Recon 3D. Using the thermodynamic properties of the compounds as constraints (see “Methods”), we estimated the Gibbs free energy for 51.3% of the 7440 reactions present in Recon 2 and 61.6% of the 10,600 reactions in Recon 3D. These constraints ensured that the reactions in the computed flux distributions operated in thermodynamically feasible directions.

### Subsystem selection to build the core (Step 2)

A proper metabolic model contains the pathways that are essential for the survival of the cell as well as the pathways that are informative of a specific metabolic behavior. In this work, we were interested in the metabolism of cancer cells. Thus, we selected as core subsystems: (a) the central carbon pathways that provide the energy, redox potential, and biomass precursors, and (b) the subsystems that have been reported to be altered in cancer cells^46–49^. Consequently, the core subsystems for our models were glycolysis, pentose phosphate pathway, citric acid cycle, oxidative phosphorylation, glutamate metabolism, serine metabolism, urea cycle, and reactive oxygen species detoxification. We have estimated the thermodynamic properties for the metabolites and the reactions in these initial subsystems. In the case of Recon 2, we provide an estimate for the Gibbs free energy of formation for 236 metabolites (94.4% of the total in the initial subsystems) and the Gibbs free energy of reaction for 143 reactions (83.1% of the reactions in the initial subsystems). In the case of Recon 3D, we provide estimated values of the thermodynamic properties for 288 metabolites (97.6%) and for 183 reactions (91.0%).

### Network expansion (Step 3)

Subsequently, to reconstruct the core network we pairwise connected the chosen subsystems using redGEM (see “Methods”). The algorithm first performed an intra-expansion of the initial subsystems. In this process, each initial subsystem was expanded to include additional reactions from the GEM whose reactants and products belong to that subsystem. These reactions can be assigned to different subsystems in the GEM which are not any of the initial subsystems and the core network would miss these additional reactions if we had considered the formal definition of the initial subsystems. The initial core subsystems of Recon 2 contained a total of 180 reactions. After the intra-expansion, 135 reactions from 21 subsystems were added. Examples of these added reactions included three from pyruvate metabolism that interconvert acetyl-CoA, acetate, malate, and pyruvate, which are all metabolites that participate in the citric acid cycle subsystem. For Recon 3D, 171 reactions from 24 subsystems were added to the 211 reactions from the initial core subsystems.

Next, the algorithm performed a directed graph search to find the reactions from the GEM that connected the subsystems for different degrees *D* (Fig. 2b and Supplementary Table 1), wherein *D* represents the distance (in number of reactions) between pairs of metabolites from the subsystems. Our final models included the connections for degree *D* = 1, that is, all the reactions that in one step connect two metabolites (excluding cofactors) belonging to any of the initial subsystems. A degree *D* = 1 was enough to pairwise connect all the initial subsystems (Fig. 2c). This resulted in a Recon 2 core network of 356 metabolites and 617 reactions and a Recon 3D core network of 440 metabolites and 796 reactions.

### Extracellular medium connection (Step 4)

Cells adapt their metabolism to the available nutrients in their extracellular environment. Consequently, a correct definition of the medium in the metabolic model is fundamental for an adequate representation of the intracellular metabolism. Given the complexity of the extracellular medium, it is particularly important to identify and classify the essentiality of the medium components and the pathways used for their metabolism. To this end, we curated the representation of the interactions of the cell with its environment into the human GEMs. First, we did not allow the exchange of intracellular metabolites lacking associated transport reactions or transport molecules containing P, CoA, or ACP (acyl carrier protein). Secondly, we allowed the synthesis of generic fatty acids from palmitate, with reactions from Recon 2 and Recon 3D (Supplementary Note 1). We next characterized the in silico minimal medium composition required for growth in the human GEMs by applying iMM (see “Methods”), which identifies the minimal set of metabolites that need to be uptaken to simulate growth. The results showed that Recon 2 required a medium with glucose, the nine essential amino acids, and some inorganics (PO_4_, NH_4_, SO_4_, O_2_), and Recon 3D simulated growth in a medium with glucose, the nine essential amino acids, the same inorganics as Recon 2, and one of the two essential fatty acids (alpha-linolenic acid and linoleic acid). The presence of the two essential fatty acids in the iMM of Recon 3D is a consequence of the improvement of the lipid metabolism^28^, where the essential fatty acids participate in the synthesis of phospholipids. This demonstrates how the algorithms and workflow can be used to compare and validate updated model reconstructions for the same organisms or between different organisms.

**Fig. 3:**
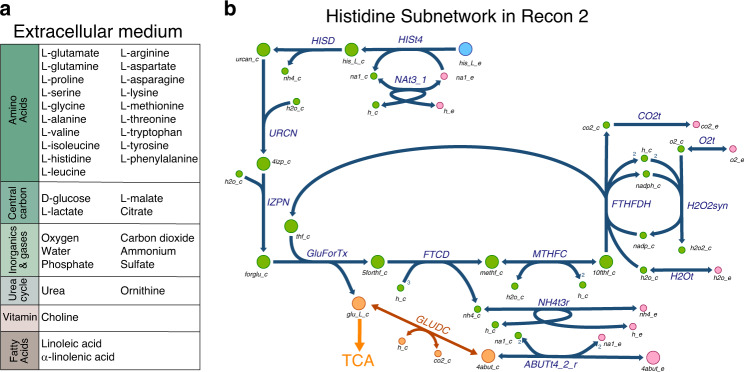
Extracellular medium utilization. **a** Extracellular medium composition defined in the models. **b** Graph of the subnetwork from Recon 2 for the uptake of l-histidine and the medium components required for its metabolism. Green represents the metabolites from the subnetwork, and orange represents the metabolites of the core network where the subnetwork is connected. In blue, the medium metabolite under study (l-histidine) and in pink, the extracellular metabolites co-utilized to metabolize l-histidine. The pathway starts with the transport of l-histidine from the extracellular space to the cytosol, where it is sequentially transformed into urocanate (urcan_c), 4-imidazolone-5-propanoate (4izp_c), N-formimidoyl-l-glutamate (forglu_c), l-glutamate (glu_L_c), 5-formiminotetrahydrofolate (5forthf_c), 5-10-methenyltetrahydrofolate (methf_c), and 10-formyltetrahydrofolate (10thf_c). 4-Aminobutanoate (4abut_c) is converted to l-glutamate through a reaction from the subsystem glutamate metabolism, and finally, l-glutamate is connected to the TCA cycle.

Seeking to identify the pathways that human cells use to uptake and secrete extracellular metabolites, we next developed the method redGEMX (see “Methods”). This algorithm finds the pathways from the GEM that are needed to connect the extracellular metabolites to the core network defined by redGEM. In this work, we considered a complex medium composition of 34 metabolites (Fig. 3a), and redGEMX found the corresponding GEM reactions that connected 26 of these extracellular metabolites (we excluded the inorganics and the fatty acids) to the core network.

An example of one of these connected metabolites is the essential amino acid l-histidine which affects many aspects of human physiology, including cognition functions and allergic reactions. The classical pathway to metabolize l-histidine consists of four steps that sequentially convert it into urocanate, 4-imidazole-5-propanoate, N-formimidoyl-l-glutamate, and ultimately, l-glutamate^50^. Interestingly, the resulting redGEMX subnetwork for l-histidine uses this classical pathway to connect it to the Recon 2 core metabolites l-glutamate and 4-aminobutanoate, both from the subsystem glutamate metabolism. The subnetwork is composed of 22 reactions, and it contains not only the classical pathway but also all the additional reactions required to balance the cofactors and by-products (Fig. 3b). These additional reactions are essential for an active main pathway, as they include the utilization of NH_4_, the sources of water and tetrahydrofolate, and the conversion of the by-product 5-formiminotetrahydrofolate to 10-formyltetrahydrofolate, which regenerates tetrahydrofolate. Cellular metabolism has evolved to give flexibility to the cells to survive and function under different conditions. This flexibility is captured in the metabolic networks with the existence of alternative pathways. For this reason, using redGEMX we found three alternative pathways of minimum size (22 reactions) to connect l-histidine to the core network of Recon 2. The alternatives emerge from the existence of different transport reactions for the extracellular metabolites. In the case of Recon 3D, l-histidine is connected to the core network using 20 reactions, and there exist two pathways of minimum size. The subnetworks connect l-histidine to the Recon 3D core metabolites l-glutamate, 5-10-methylenetetrahydrofolate, 2-oxoglutarate, and pyruvate using the classical pathway to metabolize l-histidine. The different topology of the Recon 2 and Recon 3D networks manifests in differences in the pathways used to metabolize and synthesize the compounds, thus, it is important to characterize which are the pathways used in the models. Following this approach, we added the reactions that compose all the alternative subnetworks of minimum size to the core networks to connect the 26 extracellular metabolites (Supplementary Table 2 and Supplementary Data 2).

The subnetworks generated with redGEMX provide a new perspective on the current understanding of metabolic pathways, as they not only contain the main pathway but they also include other reactions necessary to supply and consume all the cofactors and by-products. Moreover, the alternatives can be used to hypothesize which pathways cells use when growing under different conditions, such as when different nutrients are present in the environment or under different intracellular regulations when different enzymes are operational. If metabolomics data are available, the subnetworks generated with redGEMX can be classified based on pathway favorability as it has been recently done in refs. ^9,51,52^.

### Biosynthetic reactions generation (Step 5)

**Fig. 4:**
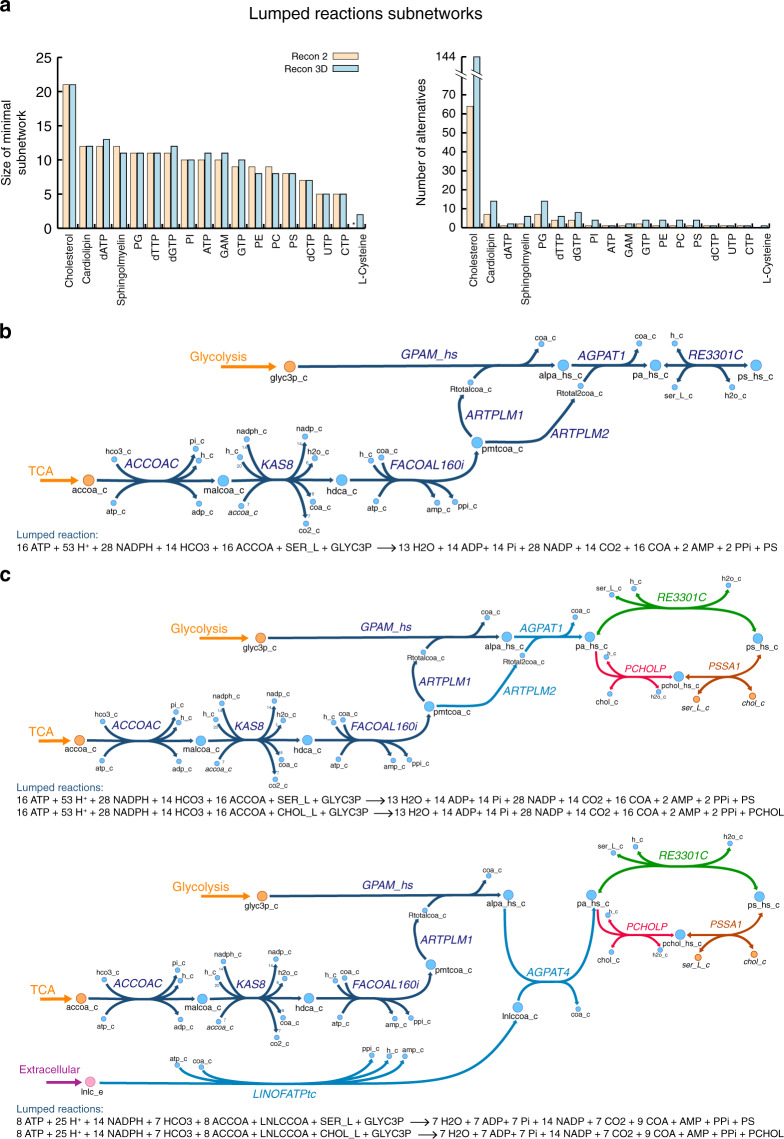
Biosynthesis of biomass building blocks. **a** Size of lumped reactions for Recon 2 and Recon 3D, and the corresponding number of alternatives to synthesize the BBBs that cannot be produced by the core nor uptaken from the extracellular medium. **b**, **c** Subnetwork for the synthesis of phosphatidylserine. Orange represents the metabolites from the core network. Blue represents the metabolites from the subnetwork for phosphatidylserine synthesis. Pink represents the extracellular metabolites. Phosphatidylserine synthesis starts from the core metabolites glycerol 3-phosphate (glyc3p_c), from glycolysis, and acetyl CoA (accoa_c), from TCA. In the first reaction, acetyl CoA is transformed into malonyl CoA (maloca_c). The next reaction (KAS8) represents the synthesis of palmitate (hdca_c) in the elongation cycle^74^. A CoA molecule is attached to palmitate to form palmitoyl CoA (pmtcoa_c), from which the two generic fatty acids are derived. These two generic fatty acids are attached to glycerol 3-phosphate to form lysophosphatidic acid (alpa_hs_c) and phosphatidic acid (pa_hs_c). Finally, serine (ser_L_c) is attached to phosphatidic acid to form phosphatidylserine (ps_hs_c). **b** Subnetwork from Recon 2 and corresponding lumped reaction. **c** The four alternative subnetworks of minimum size from Recon 3D. Phosphatidic acid can be produced with two generic fatty acids or with one generic fatty acid and the essential fatty acid linoleic acid (lnlc_e) (light blue reactions). Phosphatidylserine can be directly produced from phosphatidic acid by attaching serine (green reaction) or through the formation of phosphatidylcholine (red reaction) and then changing choline (chol_c) for serine (orange reaction).

Cellular metabolic functions, such as growth, structure maintenance, and reproduction, require the synthesis of several metabolites. In metabolic models, this is represented using the biomass reaction^53^, whose reactants, named biomass building blocks or BBBs, are the metabolites that the cell needs to survive and perform its functions. Therefore, the last step necessary for reconstructing the reduced models is the integration of the pathways necessary to synthesize the 37 BBBs that compose the defined biomass in Recon 2 and Recon 3D. Among them, 19 are uptaken directly from the extracellular medium or produced within the core network. To find the minimum number of reactions in the GEM that we need to add to the core network for the synthesis of the remaining 18 BBBs, we used lumpGEM (see “Methods”). Similarly to redGEMX, lumpGEM generates subnetworks that account for the synthesis, degradation, and balancing of all the by-products and cofactors required by the main pathway. The alternative subnetworks generated with lumpGEM can assess the flexibility of the cells to use alternative pathways to produce the BBBs, which can lead to survival in different conditions and drug resistance. Using lumpGEM, we calculated all the alternative subnetworks (set of reactions) of minimum size to capture the flexibility of the network for the biosynthesis of the BBBs (Fig. 4a, Supplementary Table 3, and Supplementary Data 3). The reactions that compose each of these subnetworks were summed up together to form an overall reaction that represented the subnetwork. These lumped reactions were then added to the core network.

The subnetworks generated with lumpGEM have the same size and number of alternatives in both Recon models for most of the BBBs, indicating that both models have the same level of flexibility for synthesizing the BBBs, with the exception of l-cysteine, dTTP and the purine nucleotides (ATP, GTP and their deoxy equivalents), cholesterol, and the phospholipids and sphingolipids. The core network of Recon 2 contains a reaction that produces l-cysteine, however, the core network of Recon 3D requires two reactions to produce it. The subnetworks that produce dTTP have the same size in both models, but a different number of alternatives. The subnetworks to produce the purine nucleotides have one more reaction and more alternatives in Recon 3D. Cholesterol is another BBB whose subnetworks agree in size for both models, but Recon 3D has more alternatives than Recon 2. The explosion of alternatives in Recon 3D is due to the parallel description of the synthesis of cholesterol in three compartments, namely cytosol, peroxisome, and endoplasmic reticulum. The differences in the lumped reactions for the phospholipids and sphingolipids between both models are due to the introduction of the essential fatty acid in their synthesis in Recon 3D.

As an example of the subnetworks that produce the BBBs, we show the synthesis of the phospholipid phosphatidylserine (Fig. 4b, c). The standard KEGG pathway^54^ for the synthesis of phosphatidylserine comprises four steps, wherein glycerol 3-phosphate is converted to lysophosphatidic acid, phosphatidic acid, CDP-diacylglycerol, and phosphatidylserine. In Recon 2, the subnetwork generated with lumpGEM for the synthesis of phosphatidylserine was composed of eight reactions. It included the KEGG pathway with the exception of the CDP-diacylglycerol intermediate, which was not connected to phosphatidylserine in the GEMs. Instead, phosphatidylserine was produced directly from phosphatidic acid by attaching serine. Additionally, the subnetwork contained the reactions required to generate from acetyl-CoA the fatty acids that would attach to glycerol 3-phosphate and to lysophosphatidic acid, which are important to consider for the final synthesis of phosphatidylserine. All the reactions involved in the synthesis of phosphatidylserine were lumped together in one reaction.

For Recon 3D, the phosphatidylserine synthesis subnetwork was generated with the same eight reactions, but in this case, four alternative subnetworks existed (Fig. 4c and Supplementary Table 4), indicating that Recon 3D has a higher flexibility in producing this BBB. The alternatives emerged from the presence of two reactions in Recon 3D that could be substituted by two other reactions in the subnetwork. One of these reactions arose from the participation of the essential fatty acid linoleate in phospholipid generation, resulting in an alternative form of synthesizing one of the tails of phosphatidic acid. Specifically, the reaction ARTPLM2, which converts palmitoyl CoA into a generic fatty acid, is not required, and instead, the essential fatty acid linoleate is transported from the extracellular medium, transformed into linoleyl-coA and attached to the lysophosphatidic acid to form phosphatidic acid. Because the core network of Recon 3D included a reaction that transforms phosphatidylcholine in phosphatidylserine, the other substitution occurred in the last step, where serine was replaced by choline and phosphatidylcholine was synthesized. The lumped reactions can be classified based on the thermodynamic favorability of their subnetworks, if metabolomics data are available, as in refs. ^9,51,52^.

The analysis performed with lumpGEM allows to characterize and classify the metabolic pathways and their alternatives, leading to an in-depth understanding of the flexibility of metabolism. In the context of GEMs, such detailed analysis of the subnetworks is often a difficult task due to their large size and interconnectivity.

By applying the redHUMAN workflow, we reconstructed four reduced metabolic models for human metabolism (Table 1). Two of them have Recon 2 as the parent GEM, and the other two are generated from the Recon 3D GEM. For both GEMs, we generated one model with the minimum set of pathways required to simulate growth, that is, one lumped reaction per BBB with subnetworks of minimum size, and another model with higher flexibility containing all the alternative pathways of minimum size required to simulate growth. The reduced models have a thermodynamic coverage of more than 92% of the compounds and more than 61% of the reactions.

**Table 1:** Statistics on the generated reduced metabolic models.

GEM	Recon 2		Recon 3D	
lumpGEM reactions	One per BBB	Smin	One per BBB	Smin
Number of metabolites	469	469	591	599
Num. enzymatic reactions	342	342	402	405
Num. boundary reactions	71	71	130	130
Num. transport reactions	946	946	1085	1092
Num. lumped reactions	15	37	15	105
Total number of reactions	1374	1396	1632	1732
Number of genes	699	699	747	748
% of metabolites with est. Gibbs energies	92.8	92.8	93.7	93.8
% of reactions with est. Gibbs energies	62.3	61.7	63.5	62.0

The models were generated from the human GEMs Recon 2 and Recon 3D. For each GEM, two reductions were performed considering either one lumped reaction per BBB (one per BBB) or all the alternatives lumped reaction with subnetworks of minimum size (Smin).

### Data integration and metabolic tasks (Step 6)

**Fig. 5:**
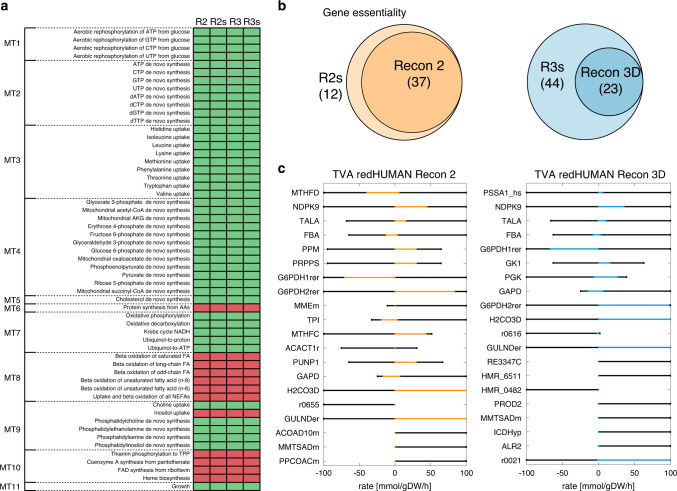
Model validation through metabolic tasks and consistency checks. **a** The 57 metabolic tasks tested in the generated reduced models. R2, R3: Recon 2, Recon 3D reduced model with one lumped reaction per BBB. R2s, R3s: Recon 2, Recon 3D reduced model with Smin. Classification of metabolic tasks in those captured by the models (green) and those not captured by the models (red). MT1: rephosphorylation of nucleoside triphosphates, MT2: de novo synthesis of nucleotides, MT3: uptake of essential amino acids, MT4: de novo synthesis of key intermediates, MT5: de novo synthesis of other compounds, MT6: protein turnover, MT7: electron transport chain and TCA, MT8: beta oxidation of fatty acids, MT9: de novo synthesis of phospholipids, MT10: vitamins and co-factors, MT11: growth. **b** Gene essentiality of the reduced models and their corresponding GEM. R2s has 829 genes associated to reactions, 37 of which are essential both in the reduced model and in Recon 2 and 12 are essential only in the reduced model. R3s has 828 genes associated to reactions, from which 23 are essential in both the reduced model and Recon 3D. The reduced model presents an additional 44 essential genes. **c** Thermo-flux variability analysis (TVA) for reactions in the reduced models. Orange represents fluxes in the reduced Recon 2 model and blue represents fluxes in the reduced Recon 3D models. The black lines correspond to the fluxes in the GEM.

Once the reduced models were generated, we investigated the metabolic tasks captured by the reduced models and we identified how the models should be curated to recover the tasks that they could not perform. First, we sequentially tested in the generated reduced models the thermodynamically feasibility of 57 metabolic tasks defined by Agren et al.^34^. The four models captured 45 of the 57 tasks, including rephosphorylation of nucleoside triphosphates, uptake of essential amino acids, de novo synthesis of nucleotides, key intermediates and cholesterol, oxidative phosphorylation, oxidative decarboxylation, and growth (Fig. 5a).

The tasks not captured by the models encompassed the synthesis of protein from amino acids, beta oxidation of fatty acids, inositol uptake, and vitamin and co-factor metabolism. We classified the causes behind their limitation into two categories: (1) the model reconstruction, specifically the definition of the biomass, or (2) the reduction properties, that is, the subsystems included in the reduction and the representation of parts of the network as lumped reactions. To recover these tasks such that they are captured by the model, the following actions should be performed: the synthesis of proteins from amino acids and vitamin and co-factor metabolism can be recovered by modifying the biomass to account for their synthesis and utilization; the inclusion of lipid metabolism subsystems can recover the beta oxidation of fatty acids; and finally, the utilization of inositol can be recovered by adding the explicit reactions that compose the subnetworks, as it was found to be hidden in the lumped reactions of phosphatidyl-inositol. This demonstrates that redHUMAN allows to build reduced models consistent not only with the GEM but also with the metabolic tasks, and these models are suitable for targeted modifications and expansions.

We next demonstrated how generic reduced models were used to integrate data to study disease physiology. We first integrated experimental data from the NCI60 cell lines in the reduced models to define the physiology of leukemia cells. In particular, we considered the exometabolomics of the cell lines HL-60, K-562, MOLT-4, CCRF-CEM, RPMI-8226, and SR, which correspond to leukemia^40,55^. Additionally, we limited the maximal growth to the doubling time reported for leukemia cells, which is 0.035 h^−1^, and we constrained according to literature values the maximum uptake rate of oxygen to 2 mmol·gDW^−1^·h^−1^ ^40^ and the ATP maintenance to 1.07 mmol·gDW^−1^·h^−1^ ^56^ (Supplementary Tables 5 and 6). We tested that all the models achieved the maximum growth when maximizing for the biomass reaction using TFA.

Next, to analyze the impact that the deletion of each gene had on the network, we performed in silico gene knockout by artificially removing a gene and measuring how the network was affected. The genes whose knockout prevented the synthesis of biomass could then be investigated as potential targets for limiting cell proliferation. The consistency of the workflow used to generate the reduced models ensures that they capture the essentiality from the GEM, that is, the genes that are part of the reduced models and are essential in the GEM they are also essential in the reduced model (Fig. 5b and Supplementary Tables 7 and 8). Furthermore, the reduced models allow the assignment of functionality to the essential genes using the lumped reactions. For example, the gene *GART* is associated with the enzymes phosphoribosylglycinamide formyltransferase, phosphoribosylglycinamide synthetase, and phosphoribosylaminoimidazole synthetase, which are all part of the subnetworks for the synthesis of the nucleotides ATP, GTP, dATP, and dGTP. Silencing this gene prevents the synthesis of these BBBs, and consequently, the models cannot synthesize biomass.

Finally, because the model reduction affects the flexibility of the network with respect to the GEM, we performed thermodynamic flux variability analysis (TVA) on the common reactions between the GEM and the reduced model. The top 20 reactions whose rate ranges changed the most in absolute value included reactions from glycolysis, the pentose phosphate pathway, folate metabolism, and nucleotide interconversion among others (Fig. 5c). For reactions such as phosphoglycerate kinase (PGK), transaldolase (TALA), and methenyltetrahydrofolate cyclohydrolase (MTHFC), the ranges of reaction rates in the reduced model decreased with respect to the corresponding reaction rates in the GEM. Some reactions, such as nucleoside-diphosphate kinase (NDPK9), were bidirectional in the GEM and became unidirectional in the reduced models. On the other hand, there were also reactions such as fumarase, (FUM) lactate dehydrogenase (LDHL), or ribose-5-phosphate isomerase (RPI) whose flux ranges fully agreed between the reduced model and the GEM. Interestingly, if we look at the percentage of rate flexibility change, the reactions from the initial subsystems did not experience a large relative change in their rates, with the exception of the reactions whose participants are precursors for the lumped reactions of the BBBs as their reaction rates are now constrained closer to the physiological state. A final calibration of the models is done using the transcriptomics data from the NCI data repository (https://www.ncbi.nlm.nih.gov/sites/GDSbrowser?acc=GDS4296) for the corresponding leukemia cell lines. We have identified that, in the four models presented in this study, over 99% of the enzymes with gene associations (more than 75% of the total enzymes) are expressed in the NCI60 leukemia cell lines (Supplementary Table 9). This suggests that the pathways selected for initializing and expanding the metabolic core network are highly relevant for the specific physiology, which are also consistent with the important pathways identified in the experimental and medical studies^46,48,57^.

### Physiology analysis

redHUMAN helps to navigate large human genome-scale metabolic models to explore and classify the metabolic pathways that cells use to function and survive under specific conditions. The thermodynamic curation performed in the genome-scale models guarantees that the reactions obey the laws of thermodynamics, discarding possible pathways that would not be compatible with the bioenergetics of the cell. As an example of how thermodynamics reduces the space of solutions to the thermodynamically feasible pathways, we analyzed the flux variability with and without thermodynamic constraints in the Recon 3D reduced model that has all the alternative lumped reactions of minimum size (Smin). The reactions l-glutamate 5-semialdehyde dehydratase (from arginine metabolism) and l-glutamate 5-semialdehyde:NAD+ oxidoreductase (from urea cycle) are bidirectional when flux variability is performed without thermodynamics and become unidirectional when their thermodynamic information is taken into account. Therefore, integrating thermodynamic information reduces the space of reaction directionality and the physiological solution space, and it eliminates thermodynamic infeasible reactions, excluding some pathways.

The leukemia-specific models generated in this study are powerful tools to analyze how the metabolic pathways are altered with respect to other cancer cells or normal cells. In particular, we can analyze how leukemia cells utilize the nutrients available in the microenvironment to biosynthesize the precursors required for growth and cellular functionality. As an example, we identified the minimal number of reactions that are required for the synthesis of phosphatidyl-serine in the reduced Recon 3D model with all the alternative lumped reactions of minimum size. We found that at least 76 reactions should be active for the production of phosphatidyl-serine including the interactions with the extracellular medium, i.e., for some alternatives the uptake of glucose, histidine, linoleic acid, oxygen, and phosphate, and the secretion of succinate, ammonia, carbon dioxide, and water. The main pathways active within the subnetwork of 76 reactions are glycolysis, the citric acid cycle, serine metabolism, and the electron transport chain. This type of analysis will enlighten our knowledge on how cells adapt their metabolism to the microenvironment allowing researchers to hypothesize how and why the cancer cells change their expression profile to adapt and survive.

## Discussion

For a better understanding of the altered metabolisms that accompany many human diseases, we have herein presented a workflow to generate reduced models for common human GEMs that can reduce the complexity of these systems to the relevant processes to be studied, making detailed in silico analyses of metabolic changes possible.

During the last years, there has been an increased generation of metabolomics data that better study what is happening in the physiology of cell metabolism compared to other omics data. This has created a need to expand the classical constraint-based modeling methods to include metabolomics information. Our thermodynamic formulation and application of TFA^12,51,58,59^ in redHUMAN allows to integrate endo- and exo-metabolomics in the models, constraining the concentration of the metabolites according to physiological data. The size of the model is directly related to the percentage of metabolites that need to be measured. Therefore, the continuous expansion in the size of genome-scale models increases the demand of larger sets of metabolomics, and such data are not always available. In addition, there is a community effort to expand constraint-based models to include information on enzyme abundancy relating the metabolic fluxes with enzymatic data and allowing to integrate transcriptomics and proteomics data into the models. These data are currently limited, but they can be continuously updated and integrated as they become available^60,61^.

Moreover, most of the existing methods to build context-specific models are data-driven, that is, the reduced models are extracted from a GEM by considering only the enzymes associated to highly expressed data, or literature-based pathways. Then, they include additional reactions that are required to simulate growth and cellular functions^33,34,62^. The main difficulty with these methods is the large amount of data required to fully characterize the initial set of reactions or core reactions. The lack of data could lead to unconnected parts and the impossibility to include reactions that could be important for the specific physiology, affecting the final model and the predictions.

redHUMAN reconstructs reduced models considering only the pathways of interest and their stoichiometric connectivity. The reduced models are built unbiased from the data, guaranteeing thermodynamic feasibility and consistency with the GEM and the metabolic tasks. The reduced models can then be used to construct context-specific models by integrating omics data, accommodating to also integrate partial data without sacrificing reactions from the network. Overall, the reduced size of the new models and their conceptual organization overcomes some of the main challenges in building genome-scale context-specific models as for example, the barrier of data network coverage. The reduced models generated with redHUMAN are powerful representations of the specific parts of the network, and they have promising applications as they are suitable to use with existing methods including MBA^62^, tINIT^34^, mCADRE^33^, uFBA^63^, GECKO^64^, ETFL^65^, TEX-FBA^66^, and IOMA^67^.

Based on our results, we propose the following approach to using these models as tools to explain and compare phenotypes. First, generate a reduced model around a desired set of subsystems and for a defined extracellular medium, and check that the model captures the metabolic tasks. Subsequently, build physiology-specific models by integrating experimental data into the reduced models. Then, test the consistency of the reduced network with respect to its parent GEM. Finally, integrate different sets of omics data, including expression, to compare different physiologies, such as diseased vs healthy or within several types of cancers. This approach will help to better investigate the alterations in metabolism that occur as diseases develop and progress. Moreover, the same procedure can be used to analyze systematically and consistently metabolic models for the same organism and to compare metabolic models of different organisms, enhancing our understanding of their similarities and differences.

Throughout this paper, we have considered a specific set of subsystems, a specific medium, and the biomass definition from the GEMs. In the future, the reduced models could be further expanded to include other pathways, a more complex medium, or more biomass components. To introduce new subsystems or pathways into the core network, redGEM should be run to find the pairwise connections between the added pathways and the rest of the core. For an expansion of the medium, redGEMX would find the connections necessary for using the new extracellular metabolites. In a similar manner, a further curation of the biomass reaction could increase the number of BBBs, requiring lumpGEM to be run to find the biosynthesis pathways for those compounds. If a higher consistency was required between the GEM and the corresponding reduction, we could find the reactions missing in the reduced model to satisfy that condition. Moreover, we have selected a set of metabolic tasks to test the generated reduced model based on the definition within the original GEM. However, these sets of tasks can be expanded or redefined according to the needs of the specific studies, which can be based on expert knowledge or experimental data, as done in ref. ^68^.

Furthermore, in this study, we have used metabolomics, proteomics, and growth data from the NCI60 cell lines to define a generic physiology for leukemia cells. The core networks of the reduced models are structurally the same across growth conditions and depend only on the structure of the corresponding GEMs. Therefore, these generic models are robust to variations in growth or data for the same physiology, and thus data for individual leukemia cell lines can be used without changing the workflow. However, if there are important differences in the data, for example across different physiological conditions, the authors suggest running the lumpGEM workflow with data integration and generate alternative subnetworks and lumped reactions, which in turn will capture the different flux profiles for each physiological state.

Overall, our analysis demonstrates how redHUMAN facilitates the characterization of differences in metabolic pathways across models and phenotypes.

## Methods

### Thermodynamic curation of the genome-scale models (GEMs)

The thermodynamic curation of the human GEMs Recon 2 and Recon 3D aims to include thermodynamic information, i.e., the Gibbs free energy of formation for the compounds and the corresponding error for the estimation, into the model. The workflow to obtain this information is as follows.

We first used MetaNetX (http://www.metanetx.org)^69^ to annotate the compounds of the GEMs with identifiers from SEED^70^, KEGG^54^, CHEBI^71^, and HMDB^72^. We then used Marvin (version 18.1, 2018, ChemAxon http://www.chemaxon.com) to transform the compound structures (canonical SMILES) into their major protonation states at pH 7 and to generate MDL Molfiles. We used the MDL Molfiles and the Group Contribution Method to estimate the standard Gibbs free energy of the formation of the compounds as well as the error of the estimation^59^.

Since the model for Recon 3D already incorporates the structure for 82% of the metabolites in the form of SMILES, we used those SMILES and followed the previous workflow from the point of obtaining the major forms at pH 7 using Marvin.

Furthermore, we have integrated in the models the thermodynamic properties for the compartments of human cells, including, pH, ionic strength, membrane potentials, and generic compartment concentration ranges from 10 pM to 0.1 M (Supplementary Table 10).

### Thermodynamics-based flux analysis (TFA)

TFA estimates the feasible flux and concentration space according to the laws of thermodynamics^11–13^. TFA is formulated as a mixed-integer linear programming (MILP) problem that incorporates the thermodynamic constraints to the original FBA problem. The Gibbs free energy of the elemental and charge balanced reactions is calculated as a function of the standard transformed Gibbs free energy of formation (depending on pH and ionic strength) and the concentrations of the products and reactants.

Considering a network with *m* metabolites and *n* reactions, the Gibbs free energy,$\Delta _r{\mathrm{G}}_i^\prime ,$ for reaction *i* is:

1 $$\Delta _r{\mathrm{G}}_i^\prime = \mathop {\sum }\limits_{j = 1}^m n_{i,j}\Delta _f{\mathrm{G}}_j^{\prime o} + RT\ln \left( {\mathop {\prod }\limits_{j = 1}^m x_j^{n_{i,j}}} \right),$$

where $i = 1, \ldots ,n,j = 1, \ldots ,m.$ *n*_*i,j*_ is the stoichiometric coefficient of compound *j* in reaction *i*; $\Delta _f{\mathrm{G}}_j^{\prime o}$ is the standard Gibbs free energy of formation of compound *j*; *x*_*j*_ is the concentration of the compound *j*, *R* is the ideal gas constant, $R = 8.31 \cdot 10^{ - 3}\frac{{\mathrm{KJ}}}{{\mathrm{K}}\;{\mathrm{mol}}}$, and *T* is the temperature. In this case, *T* = 298 K.

The value of the Gibbs free energy determines the directionality of the corresponding reaction and the thermodynamically feasible pathways. With this formulation, we included the concentrations of the metabolites as variables in the mathematical formulation. TFA allows the integration of metabolomics data into the model.

### Characterizing the extracellular in silico minimal media (iMM)

iMM is formulated as a MILP problem that introduces new variables and constraints to the TFA problem to find the minimum set of extracellular metabolites necessary to simulate growth or a specific metabolic task with the GEM^37,38^. iMM identifies the minimum number of boundary reactions (uptakes and secretions) that need to be active. The method defines new binary variables in the TFA problem that represent the state of each boundary reaction, active or inactive. New constraints link the new binary variables to the corresponding reaction rates such that if the reaction is inactive, then it should not carry flux. The objective of the problem is to maximize the number of inactive reactions.

Assuming a network with *m* metabolites and *n* reactions, the mathematical formulation of the iMM problem is the following:

2 $$\begin{array}{*{20}{c}} {\mathrm{objective}}\;{\mathrm{function}} & {\max \mathop {\sum }\limits_{k = 1}^{n_b} z_k} & {} \\ {} & {} & {} \\ {} & {{\mathrm{subject}}\;{\mathrm{to}}} & {} \\ {\mathrm{FBA}}\;{\mathrm{constraints}} & {} & {{\mathbf{S}} \cdot {\boldsymbol{v}} = 0,} \\ {} & {} & {{\boldsymbol{v}}_L \le {\boldsymbol{v}} \le {\boldsymbol{v}}_U,} \\ {\mathrm{TFA}}\;{\mathrm{constraints}} & {} & {\Delta _r{\mathrm{G}}_i^{\prime} = \mathop {\sum }\limits_{j = 1}^m n_{i,j}\Delta _f{\mathrm{G}}_j^{\prime o} + RT\ln \left( {\mathop {\prod }\limits_{j = 1}^m x_j^{n_{i,j}}} \right),i = 1, \ldots ,n,} \\ {} & {} & {\Delta _r{\mathrm{G}}_i^\prime - M + M \cdot b_i^F \le 0} \\ {} & {} & { - \Delta _r{\mathrm{G}}_{i}^{\prime} - M + M \cdot b_i^R \le 0} \\ {} & {} & {v_i^{F,R} - M \cdot b_i^{F,R} \le 0} \\ {} & {} & {b_i^F + b_i^R \le 1} \\ {\mathrm{IMM}}\;{\mathrm{constraints}} & {} & {{\mathbf{b}}^F + {\mathbf{b}}^R + C \cdot {\mathbf{z}} \le C,} \end{array}$$

where *n*_*b*_ is the total number of boundary reactions in the model, *z*_*k*_ are new binary variables for all the boundary reactions, **S** is the stoichiometric matrix, ***v*** are the net fluxes for all the reactions and $v_i^F,v_i^R$ are the corresponding net-forward and net-reverse fluxes, so that, $v_{i} = v_{i}^{F} - v_{i}^{R},\;\;{\mathrm{for}}\;{\mathrm{all}}\;\; i = 1, \ldots ,n$. ${\boldsymbol{v}}_L$ and ${\boldsymbol{v}}_U$ are the lower and upper bound, respectively, for all the reactions in the network. $\Delta _r{\mathbf{G}}^\prime$ is the Gibb’s free energy of the reactions defined in TFA. ${\mathbf{b}}_{}^F$ and ${\mathbf{b}}_{}^R$ are the binary variables for the forward or reverse fluxes of all the reactions (coupled to TFA). *M* is a big constant (bigger than all upper bounds) and *C* is an arbitrary large number. In this case, if $z_k = 0$, then reaction *k* is active.

### redGEM, redGEMX, and lumpGEM

The redGEM, redGEMX, and lumpGEM algorithms seek to generate systematic reductions of the GEMs starting from chosen subsystems (or lists of reactions and metabolites, such as the synthesis pathway of a target metabolite), based on the studied physiology and the specific parts of the metabolism that are of interest.

redGEM is a published algorithm^35^ that extracts the reactions that pairwise-connect the initial subsystems from the GEM, generating a connected network named the core network.

The inputs for redGEM are (i) the GEM, (ii) the starting subsystems or an initial set of reactions, (iii) the extracellular medium metabolites, (iv) a list with the GEM cofactor pairs, and (v) the desired degree of connectivity. The algorithm then performs an expansion (by graph search) of the starting subsystems by finding the reactions that pairwise-connect the subsystems up to the selected degree (see ref. ^35^ for further details). For example, for a degree equal to 2, it will connect the metabolites from the starting subsystem that are one and two reactions away in the GEM.

**Fig. 6:**
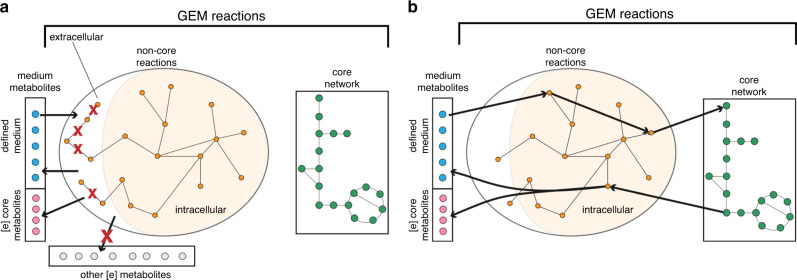
redGEMX method. **a** Classification of the reactions from the GEM into core (green) and non-core reactions (orange), and classification of the extracellular metabolites from the GEM into those that are part of the medium that we want to connect (blue), those that are present in the core (pink), and the others (gray). The algorithm will block the non-core reactions that involve only extracellular metabolites as well as the boundary and transport reactions of the metabolites that are not part of the medium (gray). **b** The algorithm finds the minimal set of reactions that are required to connect each of the medium metabolites (blue) to the core network, uses the core network to balance the reactions, and secretes metabolites from the medium (blue or pink).

redGEMX is a formulated algorithm that finds the pathways in the GEM that connect the extracellular medium to the core network generated with redGEM (Fig. 6). These pathways are added to the core network.

The redGEMX method involves five steps:

1. Classify the extracellular metabolites of the GEM into 3 classes:
  - Those that are part of the medium that we want to connect,
  - Those that are already present in the inter-connected subsystems network,
  - Those that do not belong to (a) nor (b).
2. Classify the reactions from the GEM into 2 classes:
  - Those that belong to the inter-connected subsystems network (core-reactions),
  - those that do not belong to the inter-connected subsystems network (non-core reactions).
3. Block the flux through the reactions in the GEM that involve only extracellular metabolites.
4. Block the flux through the boundary reactions of other metabolites in the GEM (1c). Steps (3) and (4) guarantee that the subnetwork reaches the core network.
5. Force the uptake of a medium metabolite (1a, one-by-one) and minimize the number of non-core reactions (2b) required to connect this extracellular metabolite to any core metabolite participating in a core reaction (2a). Note that the subnetwork will contain any reaction required to balance the by-products secreted by the subnetwork and/or the core network.

The redGEMX is a MILP problem that is formulated as follows:

(i) Consider the TFA problem of the model that we want to reduce.
(ii) Create binary variables *z*_*i*_ for each non-core reaction (2b). Non-core reactions are denoted as *R*^*nc*^.
(iii) Generate a constraint that controls the flux for each non-core reaction:
  3 $${\mathbf{b}}^F + {\mathbf{b}}^R + {\mathbf{z}} \le 1,$$
  where **b**^*F*^ and **b**^*R*^ are the binary variables for the forward and reverse fluxes of all the reactions (coupled to the TFA constraints); when *z*_*i*_ = 1, the corresponding reaction is inactive.
(iv) Build the following MILP problem for each extracellular medium metabolite (1a)
  4 $$\max \mathop {\sum }\limits_{i = 1}^{R^{nc}} z_i$$
  subject to:
  5 $${\mathbf{b}}^F + {\mathbf{b}}^R + {\mathbf{z}} \le 1,$$
  6 $$v_{eM,j} \ge c,$$
  where *v*_*eM,j*_ is the flux of the *j*th extracellular medium metabolite (1a), and *c* is a small number.

lumpGEM is a published algorithm^36^ that generates elementally balanced lumped reactions for the synthesis of the biomass building blocks (BBBs). Using a MILP formulation, lumpGEM identifies the smallest subnetwork (minimum number of reactions from the GEM) required to produce each BBB from metabolites that belong to the core network using reactions from the GEM that are not part of the core. With this formulation, we can identify all the alternative subnetworks (of minimal size or larger) for the synthesis of each BBB (one by one). lumpGEM generates, for each BBB, an overall lumped reaction by adding all the reactions that constitute each subnetwork (see ref. ^36^ for further details). Note here, different subnetworks can give rise to the same overall lumped reaction. This implies that although we produce all the alternative subnetworks with their associated lumped reactions, only the unique lumped reactions will be added to the final reduction.

### Software

The simulations of this article have been done with Matlab 2017b and CPLEX 12.7.1. Escher^73^ has been used to draw the subnetworks in the figures.

### Reporting summary

Further information on research design is available in the Nature Research Reporting Summary linked to this article.

## Supplementary information

Supplementary Information

Supplementary Data 1

Supplementary Data 2

Supplementary Data 3

Reporting Summary

Description of Additional Supplementary Files

## Data Availability

The models generated in this work and the data integrated in the models to define the physiology of leukemia cells are available under the APACHE 2.0 license at https://github.com/EPFL-LCSB/redhuman.

## Code availability

The scripts to generate the results for this paper are available under the APACHE 2.0 license at https://github.com/EPFL-LCSB/redhuman. The code for TFA is available at https://github.com/EPFL-LCSB/mattfa. The code to reduce the human GEMs (redGEM), to connect the extracellular medium to the core (redGEMX), and to generate the biosynthetic lumped reactions (lumpGEM) are available at https://github.com/EPFL-LCSB/redgem.
