# Author Correction: Analysis of human metabolism by reducing the complexity of the genome-scale models using redHUMAN

**DOI:** 10.1038/s41467-020-17694-4

**Published:** 2020-07-23

**Authors:** Maria Masid, Meric Ataman, Vassily Hatzimanikatis

**Affiliations:** 1 Laboratory of Computational Systems Biotechnology, École Polytechnique Fédérale de Lausanne (EPFL), Lausanne, Switzerland; 2 Computational and Systems Biology, Biozentrum, University of Basel, Basel, Switzerland

**Keywords:** Metabolic engineering, Cancer metabolism, Metabolic pathways, Computational models, Data integration

Correction to: *Nature Communications* 10.1038/s41467-020-16549-2, published online 4 June 2020.

The original version of this Article’s Acknowledgement section incompletely read:

This project has received financial support from the European Union’s Horizon 2020 research and innovation programme under the Marie Skłodowska Curie Grant Agreement No. 675585 SyMBioSys and under Grant Agreement No. 686070.

Instead of:

We would like to thank Dr. G. Fengos, Dr. J.P. Vieira and Dr. D.R. Weilandt for constructive discussions and feedback; and Dr. K. Butler for valuable comments proofreading this paper. This project has received financial support from the European Union’s Horizon 2020 research and innovation programme under the Marie Skłodowska Curie Grant Agreement No. 675585 SyMBioSys, and under Grant Agreement No. 686070, and from the RTD project MicroScapeX (grant 2013/158) funded by SystemsX.ch, the Swiss Initiative for Systems Biology evaluated by the Swiss National Science Foundation.

This has now been corrected in both the PDF and HTML versions of the Article.

