# Supplementary material for: Analysis of human metabolism by reducing the complexity of the genome-scale models using redHUMAN
Source: Nat Commun. 2020 Jun 4;11:2821. doi: 10.1038/s41467-020-16549-2 (PMC7272419; doi:10.1038/s41467-020-16549-2)
Supplement: Supplementary file 1 — Supplementary Information [file 41467_2020_16549_MOESM1_ESM.pdf]

Supplementary Information

Analysis of human metabolism by reducing the complexity of the genome-scale models using redHUMAN

Masid, et al.

Supplementary Notes

Supplementary Note 1: Curation of the generic lipids in human GEMs. We curated the synthesis of the generic fatty acids in both models. Some compounds from the lipid metabolism of the human GEMs contain R-groups as generic compounds representing fatty acids with different chain lengths. The biosynthesis of fatty acids starts with the conversion of acetyl-coA into malonyl-coA. Next, the primary fatty acid synthesized is palmitate (C16:0) that can later enter the elongation cycle to produce fatty acids with longer chains. We connected palmitate to the R-groups using reactions already defined in the original Recon 2 and Recon 3D models. These reactions substitute the elongation process and define directly the generic fatty acids (R-groups).

Supplementary Tables

Supplementary Table 1: redGEM statistics. Expansion of the starting subsystems by pairwise connections for different degrees. D = 0 does not include interconnections across subsystems, only the intra-expansion of the initial subsystems

	Recon 2				Recon 3D			
Degree of connection	D = 0	D = 1	D = 2	D = 3	D = 0	D = 1	D = 2	D = 3
Number of metabolites	254	356	434	487	300	440	530	592
Number of reactions	346	617	1286	1412	416	796	1451	1620

Supplementary Table 2: redGEMX connections. Size of subnetworks [and number of alternatives] to connect the medium metabolites to the core subsystems. As an example, the two last columns show the core metabolites and the subsystems to which the extracellular connects for one alternative.

		Extracellular metabolites	Recon 2 Num. of reactions [num of alternatives]	Recon 3D Num. of reactions [num of alternatives]
Amino acids	Glutamate Family	L-glutamate	10 [8]	9 [1]
		L-glutamine	11 [3]	11 [4]
		L-proline	13 [1]	12 [1]
		L-arginine	11 [3]	8 [1]
	Serine Family	L-serine	6 [1]	6 [5]
		glycine	18 [1]	8 [21]
	Pyruvate Family	L-alanine	12 [27]	9 [6]
		L-valine	24 [57]	23 [3]
		L-isoleucine	27 [3]	23 [2]
		L-leucine	32 [1]	23 [1]
	Aspartate Family	L-aspartate	13 [1]	7 [4]
		L-asparagine	14 [2]	9 [1]
		L-lysine	33 [13]	40 [33]
		L-methionine	42 [1]	22 [4]
		L-threonine	21 [1]	6 [1]
	Aromatic Family	L-tryptophan	39 [8]	38 [1]
		L-tyrosine	27 [3]	25 [1]
		L-phenylalanine	27 [2]	27 [1]
		L-histidine	22 [3]	20 [2]
Central carbon compounds		D-glucose	5 [1]	5 [1]
		L-lactate	5 [1]	5 [1]
		L-Malate	13 [2]	9 [5]
		Citrate	10 [2]	5 [9]
Vitamin		Choline	14 [10]	11 [7]
Urea cycle products		Ornithine	7 [3]	7 [3]
		Urea	7 [3]	7 [3]

Supplementary Table 3: Lumped reactions for Recon 2 and Recon 3D. Size of lumped reactions for Recon 2 and Recon 3D and corresponding number of alternatives. The rest of the BBBs either are directly uptaken (available in the medium) or they can be produced in the core network.

	Biomass building blocks	Recon 2		Recon 3D	
		Network size	Number alternatives	Network size	Number alternatives
Amino acid	L-Cysteine	Produced by the core	Produced by the core	2	1
Nucleotides	ATP	10	1	11	1
	GTP	9	2	10	4
	CTP	5	1	5	1
	UTP	5	1	5	1
	dGTP	11	4	12	8
	dCTP	7	1	7	1
	dATP	12	1	13	2
	dTTP	11	4	11	6
Lipid	Cholesterol	21	64	21	144
Phospholipids & Sphingolipids	1-Phosphatidyl-1D-Myo-Inositol	10	1	10	4
	Phosphatidylserine	8	1	8	4
	Phosphatidylcholine	9	1	8	4
	Phosphatidylethanolamine	9	1	8	4
	Phosphatidylglycerol	11	7	11	14
	Cardiolipin	12	7	12	14
	Sphingomyelin	12	2	11	6
	Growth Associated Maintenance	10	1	11	2

Supplementary Table 4: Lumped reactions for phosphatidylserine in Recon 3D. Stoichiometry of the alternative lumped reactions generated with lumpGEM for the biomass building block phosphatidylserine in Recon 3D.

	Lumped reactions for phosphatidylserine in Recon 3D			
	Lumped Reaction 1	Lumped Reaction 2	Lumped Reaction 3	Lumped Reaction 4
ATP	-16	-16	-8	-8
H^+	-53	-53	-25	-25
NADPH	-28	-28	-14	-14
HCO3	-14	-14	-7	-7
ACCOA	-16	-16	-8	-8
SER_L	-1	0	-1	0
CHOL	0	-1	0	-1
LNLCCOA	0	0	-1	-1
GLYC3P	-1	-1	-1	-1
H2O	13	13	7	7
ADP	14	14	7	7
Pi	14	14	7	7
NADP	28	28	14	14
CO2	14	14	7	7
COA	16	16	9	9
AMP	2	2	1	1
PPi	2	2	1	1
PS	1	0	1	0
PCHOL	0	1	0	1
	53 H^+ + 16 ATP + 28 NADPH + 14 HCO3 + 16 ACCOA + SER_L + GLYC3P -> 13 H2O + 14 ADP + 14 Pi + 28 NADP + 14 CO2 + 16 COA + 2 AMP + 2 PPi + PS	53 H^+ + 16 ATP + 28 NADPH + 14 HCO3 + 16 ACCOA + CHOL + GLYC3P -> 13 H2O + 14 ADP + 14 Pi + 28 NADP + 14 CO2 + 16 COA + 2 AMP + 2 PPi + PCHOL	25 H^+ + 8 ATP + 14 NADPH + 7 HCO3 + 8 ACCOA + SER_L + LNLCCOA + GLYC3P -> 7 H2O + 7 ADP + 7 Pi + 14 NADP + 7 CO2 + 9 COA + AMP + PPi + PS	25 H^+ + 8 ATP + 14 NADPH + 7 HCO3 + 8 ACCOA + LNLCCOA + CHOL + GLYC3P -> 7 H2O + 7 ADP + 7 Pi + 14 NADP + 7 CO2 + 9 COA + AMP + PPi + PCHOL

Supplementary Table 5: Leukemia physiology. Extracellular data integrated in the models to constrain the intake and secretion of extracellular metabolites.

	Reaction in model	Lower bound	Upper bound
Citrate	EX_cit_e	-0.0007	0.0015
Malate	EX_mal_L_e	0.0002	0.0012
Choline	EX_chol_e	-0.0078	-0.0004
Ornithine	EX_orn_e	0.0077	0.0498
Alanine	EX_ala_L_e	-0.0137	0.1695
Arginine	EX_arg_L_e	-0.0752	-0.0090
Asparagine	EX_asn_L_e	-0.0151	-0.0035
Aspartate	EX_asp_L_e	-0.0098	0.0051
Glucose	EX_glc_e	-3.3230	-0.4623
Glutamate	EX_glu_L_e	0.0089	0.0883
Glutamine	EX_gln_L_e	-0.6398	-0.1672
Glycine	EX_gly_e	-0.0031	0.0139
Isoleucine	EX_ile_L_e	-0.0420	-0.0083
Lactate	EX_lac_L_e	0.5531	3.7791
Leucine	EX_leu_L_e	-0.0508	-0.0099
Lysine	EX_lys_L_e	-0.0697	-0.0170
Phenylalanine	EX_phe_L_e	-0.0206	-0.0045
Proline	EX_pro_L_e	-0.0015	0.0145
Serine	EX_ser_L_e	-0.1140	-0.0262
Threonine	EX_thr_L_e	-0.0451	-0.0092
Tryptophan	EX_trp_L_e	-0.0054	-0.0008
Tyrosine	EX_tyr_L_e	-0.0276	-0.0054
Valine	EX_val_L_e	-0.0483	-0.0098
Methionine	EX_met_L_e	-0.0186	-0.0041
Oxygen	EX_o2_e	-2	0
Growth	biomass	0	0.0354
ATP maintenance	ATPM	1.07	100

Supplementary Table 6: Leukemia physiology. Extracellular data integrated in the models to constrain the extracellular concentrations of the metabolites.

	Variable in model	Lower bound	Upper bound
3-Hydroxyanthranilate	LC_3hanthrn_e	-17.6225	-17.5215
4-Aminobutanoate	LC_4abut_e	-15.6566	-12.998
Acetoacetate	LC_acac_e	-14.0479	-13.567
Adenosine	LC_adn_e	-100	-16.5006
S-Adenosyl-L-Homocysteine	LC_ahcys_e	-17.1933	-16.2063
2-Oxoglutarate	LC_akg_e	-15.6860	-15.1312
L-Alanine	LC_ala_L_e	-10.9925	-7.6359
L-Arginine	LC_arg_L_e	-6.9545	-6.8303
L-Asparagine	LC_asn_L_e	-8.0950	-7.9203
L-Aspartate	LC_asp_L_e	-8.8353	-8.6895
Choline	LC_chol_e	-10.8158	-10.5017
Citrate	LC_cit_e	-12.2716	-11.4708
Creatine	LC_creat_e	-10.8187	-10.6980
L-Carnitine	LC_crn_e	-14.1057	-13.8208
Fumarate	LC_fum_e	-13.4448	-13.1200
L-Glutamine	LC_gln_L_e	-7.6293	-6.5510
L-Glutamate	LC_glu_L_e	-8.6402	-8.1777
Glycine	LC_gly_e	-8.8527	-8.6086
Guanidinoacetic Acid	LC_gudac_e	-9.6591	-9.1620
L-Homocysteine	LC_hcys_L_e	-15.9947	-15.0187
3-Hydroxy-L-Kynurenine	LC_hLkynr_e	-19.5847	-18.4311
Isocitric Acid	LC_icit_e	-15.1765	-14.8061
L-Isoleucine	LC_ile_L_e	-8.1052	-7.9730
L-2-Aminoadipate	LC_L2aadp_e	-15.9991	-15.4878
(S)-Lactate	LC_lac_L_e	-5.0995	-4.6995
L-Kynurenine	LC_Lkynr_e	-15.3602	-14.7654
L-Lysine	LC_lys_L_e	-9.1042	-8.4909
L-Homoserine	LC_hom_L_e	-10.6130	-10.2175
(S)-Malate	LC_mal_L_e	-12.2526	-11.9394
L-Methionine	LC_met_L_e	-9.9144	-9.3876
Ornithine	LC_orn_e	-8.9184	-8.5935
L-Phenylalanine	LC_phe_L_e	-10.0679	-9.5613
L-Serine	LC_ser_L_e	-10.6420	-8.7444
Spermidine	LC_spmd_e	-15.2091	-14.8503
Succinate	LC_succ_e	-11.2943	-11.1637
Taurine	LC_taur_e	-13.1674	-12.0361
L-Threonine	LC_thr_L_e	-9.45495	-8.9928
L-Tryptophan	LC_trp_L_e	-11.2214	-10.7397
L-Tyrosine	LC_tyr_L_e	-9.2399	-8.9257
L-Valine	LC_val_L_e	-9.4606	-9.0726

Supplementary Table 7: Gene essentiality analysis. List of genes that are essential in the reduced Recon 2 models, and the corresponding reactions associated to these genes. The third column indicates if the gene is essential in the GEM.

GENE	REACTIONS	Essential in GEM
1738.1	GCC2am I GCC2bim I GCC2cm I GCCam I GCCbim I GCCcm I PDHm I r1154	-
2531.1	3DSPHR	Yes
2194.1	KAS8	Yes
471.1	AICART I IMPC	Yes
10606.1	AIRCr I PRASCS	Yes
790.1	CBPS I ASPCTr I DHORTS	Yes
7108.1	C14STRr I r0780	Yes
50814.1	C3STDH1Pr I C4STMO2Pr I C3STDH1r	Yes
3295.1	C3STKR2r	Yes
6307.1	C4STMO1r	Yes
54675.1	CLS_hs	Yes
875.1	CYSTS	Yes
51727.1	UMPK I UMPK2 I UMPK3 I UMPK4 I UMPK5 I UMPK7 I CYTK8 I CYTK6 I CYTK7 I CYTK1 I CYTK10	Yes
2987.1	GK1	-
1718.1	r0783 I DSREDUCr I r1380	-
1717.1	DHCR72r I DHCR71r	Yes
1719.1	DHFR I r0224	Yes
1723.1	DHORD9	Yes
9453.1	DMATT I GRTT	-
4597.1	DPMVDc	Yes
1841.1	DTMPK I NDP8	-
10682.1	EBP1r I EBP2r I r1381	Yes
2819.1	G3PD1 I r0202	-
2618.1	r0666	Yes
5471.1	GLUPRT	Yes
3156.1	r0488 I HMGCOARc	Yes
4047.1	LNSTLSr	Yes
4598.1	MEVK1c	Yes
51477.1	MI1PS	Yes
7372.1	OMPDC I ORPT	Yes
5338.1	RE3273C I RE3301C	-
114971.1	PGPP_hs	Yes
9489.1	PGPPT	Yes
10654.1	PMEVKc	Yes
5198.1	PRFGS	Yes
23761.1	PSDm_hs	-
9791.1	PSSA1_hs	-
6240.1	r0472 I r0474 I r0475	-
6241.1	r0472 I r0474 I r0475	-
50484.1	r0472 I r0474 I r0475	-
22934.1	RPI I r0249	Yes
10558.1	SERPT	Yes
9517.1	SERPT	Yes
55304.1	SERPT	Yes
259230.1	SMS	Yes
6713.1	SQLEr	Yes
2222.1	SQLSr	Yes
7298.1	TMDS	Yes
1595.1	r0781	Yes

Supplementary Table 8: Gene essentiality analysis. List of genes that are essential in the in the reduced Recon 3D models, and the corresponding reactions associated to these genes. The third column indicates if the gene is essential in the GEM.

GENE	REACTIONS	Essential in GEM
1738.1	GCC2am I GCC2bim I GCC2cm I GCCam I GCCbim I GCCcm I PDHm I r1154	-
8050.1	2OXOADOXm I AKGDm I PDHm	-
1743.1	2OXOADOXm I AKGDm	-
2531.1	3DSPHR	Yes
10606.1	AIRCr I PRASCS	-
790.1	CBPS I ASPCTr I DHORTS	Yes
7108.1	C14STRr	-
50814.1	C3STDH1Pr I C4STMO2Pr I C3STDH1r	Yes
54675.1	CLS_hs	Yes
51727.1	UMPK2 I UMPK3 I UMPK4 I UMPK5 I UMPK7 I UMPK I CYTK1 I CYTK10 I CYTK6 I CYTK8 I CYTK7	-
1717.1	DHCR72r I DHCR71r	-
1723.1	DHORD9	Yes
4597.1	DPMVDc I DPMVDx	Yes
10682.1	EBP1r I EBP2r	Yes
2194.1	KAS8	-
1719.1	DHFR I r0224	Yes
2618.1	r0666	-
3158.1	HMGCOASim	-
4047.1	LNSTLSr	Yes
4598.1	MEVK1c I MEVK1x	Yes
51477.1	MI1PS I HMR_6572	Yes
1841.1	DTMPK I NDP8	-
7372.1	ORPT I OMPDC	Yes
114971.1	PGPP_hs	-
9489.1	PGPPT	Yes
10654.1	PMEVKc I PMEVKx	Yes
10558.1	SERPT	Yes
9517.1	SERPT	Yes
55304.1	SERPT	Yes
259230.1	SMS	-
6713.1	SQLEr	Yes
2222.1	SQLSr	Yes
2819.1	r0202 I G3PD1 I HMR_0478	Yes
875.1	CYSTS	Yes
22934.1	r0249 I RPI	-
50484.1	r0472 I r0474 I r0475	-
6240.1	r0472 I r0474 I r0475	-
6241.1	r0472 I r0474 I r0475	-
3156.1	r0488 I HMR_4630	Yes
1595.1	r0781	-
2987.1	GK1	-
471.1	AICART I IMPC	-
5471.1	GLUPRT	-
5198.1	PRFGS	-
7298.1	TMDS	Yes
7384.1	CYOR_u10mi I CYOOm2i	-
7388.1	CYOR_u10mi I CYOOm2i	-
4519.1	CYOR_u10mi I CYOOm2i	-
10975.1	CYOR_u10mi I CYOOm2i	-
7385.1	CYOR_u10mi I CYOOm2i	-
7386.1	CYOR_u10mi I CYOOm2i	-
1537.1	CYOR_u10mi I CYOOm2i	-
27089.1	CYOR_u10mi I CYOOm2i	-

7381.1	CYOR_u10mi I CYOOm2i	-
1351.1	CYOOm3i I CYOOm2i	-
1347.1	CYOOm3i I CYOOm2i	-
1329.1	CYOOm3i I CYOOm2i	-
1327.1	CYOOm3i I CYOOm2i	-
341947.1	CYOOm3i I CYOOm2i	-
1350.1	CYOOm3i I CYOOm2i	-
1349.1	CYOOm3i I CYOOm2i	-
1339.1	CYOOm3i I CYOOm2i	-
1345.1	CYOOm3i I CYOOm2i	-
9377.1	CYOOm3i I CYOOm2i	-
170712.1	CYOOm3i I CYOOm2i	-
1340.1	CYOOm3i I CYOOm2i	-
1337.1	CYOOm3i I CYOOm2i	-

Supplementary Table 9: Reactions with gene expression. Number of reactions associated to expressed genes in the corresponding NCI60 leukemia cell lines.

MODEL	Reduced Recon2	Reduced Recon2 Smin	Reduced Recon3D	Reduced Recon3D Smin
Total number of reactions	1429	1451	1691	1738
Number of reactions with GPRs	1194	1215	1282	1317
Number of leukemia NCI60 expressed reactions	1190	1211	1281	1316
% of expressed reactions w.r.t. total number of reactions	83.28%	83,46%	75.75%	75.72%
% of expressed reactions w.r.t. number of reactions with GPRs	99.66%	99.67%	99.92%	99.92%

Supplementary Table 10: Thermodynamic parameters. Thermodynamic properties used for the compartments in the models.

<table>
<tr><th>Compartment</th><th>pH</th><th>Δ Ψ [mV]</th><th>Ionic Strength [M]</th><th>Concentrations range [M]</th></tr>
<tr><td>cytosol</td><td>7.2</td><td>0</td><td rowspan="10">0.15</td><td rowspan="9">[10^{-11} – 0.08]</td></tr>
<tr><td>mitochondria</td><td>8</td><td>-155</td></tr>
<tr><td>Inner-mitochondria membrane space (*)</td><td>7.2</td><td>0</td></tr>
<tr><td>vacuole</td><td>7</td><td>0</td></tr>
<tr><td>peroxisome</td><td>7</td><td>12</td></tr>
<tr><td>Golgi apparatus</td><td>6.35</td><td>0</td></tr>
<tr><td>endoplasmic reticulum</td><td>7.2</td><td>0</td></tr>
<tr><td>nucleus</td><td>7.2</td><td>0</td></tr>
<tr><td>lysosome</td><td>4.7</td><td>19</td></tr>
<tr><td>extracellular</td><td>7.4</td><td>30</td><td>[10^{-11} – 0.1]</td></tr>
</table>

(*) only the Recon 3D models have this compartment

References for pH values:

- Casey et al. *Sensors and regulators of intracellular pH*. Nature Reviews, 2010
- Alberts et al. *Molecular Biology of the cell*. 4th ed New York: Garland Science, 2002

Reference for membrane potential and ionic strength:

- Haraldsdóttir et al. *Quantitative Assignment of Reaction Directionality in a Multicompartmental Human Metabolic Reconstruction*. Biophysical Journal, 2012
