# Supplementary material for: Analysis of human metabolism by reducing the complexity of the genome-scale models using redHUMAN
Source: Nat Commun. 2020 Jun 4;11:2821. doi: 10.1038/s41467-020-16549-2 (PMC7272419; doi:10.1038/s41467-020-16549-2)
Supplement: Supplementary file 6 — Description of Additional Supplementary Files [file 41467_2020_16549_MOESM6_ESM.pdf]

Description of Additional Supplementary Files

File Name: Supplementary Data 1
Description: Thermodynamic data for Recon 2 and Recon 3D. Gibbs free energy of formation for the compounds, computed with the group contribution method.

File Name: Supplementary Data 2
Description: Subnetworks to connect the extracellular medium in Recon 2 and Recon 3D. Subnetworks generated with redGEMX to connect the metabolites from the extracellular medium to the core network.

File Name: Supplementary Data 3
Description: Lumped reactions for the synthesis of biomass building blocks for Recon 2 and Recon 3D. Overall lumped reactions generated with lumpGEM to biosynthesize the biomass building blocks.
